# Reduced‐Dose Dabrafenib–Trametinib for BRAF V600E–Mutant Lung Adenocarcinoma in a Very Elderly Patient With ECOG PS 2

**DOI:** 10.1002/rcr2.70620

**Published:** 2026-05-28

**Authors:** Shun Suyama, Keeya Sunata, Koyuru Uchibori, Shun Shinomiya, Tetsuo Tani, Saeko Takahashi

**Affiliations:** ^1^ Division of Pulmonary Medicine Saiseikai Central Hospital Tokyo Japan; ^2^ Division of Pulmonary Medicine, Department of Medicine Keio University School of Medicine Tokyo Japan

**Keywords:** BRAF V600E, dabrafenib, lung adenocarcinoma, reduced‐dose therapy, trametinib, very elderly

## Abstract

BRAF V600E mutations occur in approximately 1%–2% of non–small cell lung cancers (NSCLCs). Dabrafenib plus trametinib has demonstrated clinical efficacy in BRAF V600E–mutant NSCLC, although pyrexia frequently leads to treatment interruption. We report an 86‐year‐old woman with lung adenocarcinoma harbouring a BRAF V600E mutation and an ECOG performance status (PS) of 2. She had undergone partial lung resection (Stage IA2) 4 years earlier and later developed pleural dissemination and liver metastases. Although she initially declined systemic therapy because of concerns about treatment‐related adverse events, she subsequently experienced progressive malignant pleural effusion requiring repeated thoracentesis followed by chest tube drainage and pleurodesis, accompanied by worsening dyspnea, pleuritic chest pain and appetite loss. She then requested therapy for symptom relief and disease control. Reduced‐dose dabrafenib and trametinib were initiated with prophylactic naproxen to mitigate the risk of pyrexia. Within 2 weeks, her chest pain and appetite improved, and ECOG PS recovered to 1. Treatment has been continued for over 6 months with sustained disease stability. This case suggests that an upfront dose‐attenuation strategy combined with proactive toxicity management, including pyrexia prophylaxis, may represent a practical approach to maintain treatment continuity and clinical benefit in selected very elderly or frail patients.

## Graphical abstract

We describe a very elderly patient with ECOG performance status 2 who initially declined systemic therapy because of concerns about adverse events but later achieved symptom improvement and sustained disease control with reduced‐dose dabrafenib and trametinib.

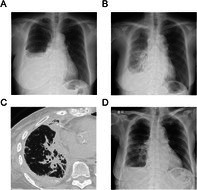

## 1 Introduction

BRAF V600E mutations represent an uncommon but clinically relevant molecular subtype of non–small cell lung cancer (NSCLC), occurring in approximately 1%–2% of cases [1]. Dabrafenib plus trametinib is an established treatment option for this subtype, with clinical trials demonstrating meaningful responses [2]. However, the regimen is associated with several treatment‐related adverse events—including frequent pyrexia, fatigue, nausea, rash and liver or cardiac dysfunction—which often necessitate dose interruption or reduction, particularly in older or frail patients [2]. Here, we describe a very elderly patient with ECOG performance status (PS) 2 who initially declined systemic therapy because of concerns about such adverse events but later achieved symptom improvement and sustained disease control with reduced‐dose dabrafenib and trametinib.

## 2 Case Report

An 86‐year‐old non‐smoking woman presented with worsening right pleuritic chest pain, dyspnea and anorexia. Her medical history included chronic kidney disease, prior cerebral infarction, hypertension, dyslipidemia, type 2 diabetes mellitus and femoral neck fracture. These comorbidities, together with cancer‐related fatigue and dyspnea, contributed to an ECOG PS of 2. Four years earlier, she had undergone partial resection of the right upper and middle lobes for stage IA2 (pT1bN0M0) lung adenocarcinoma.

**FIGURE 1:**
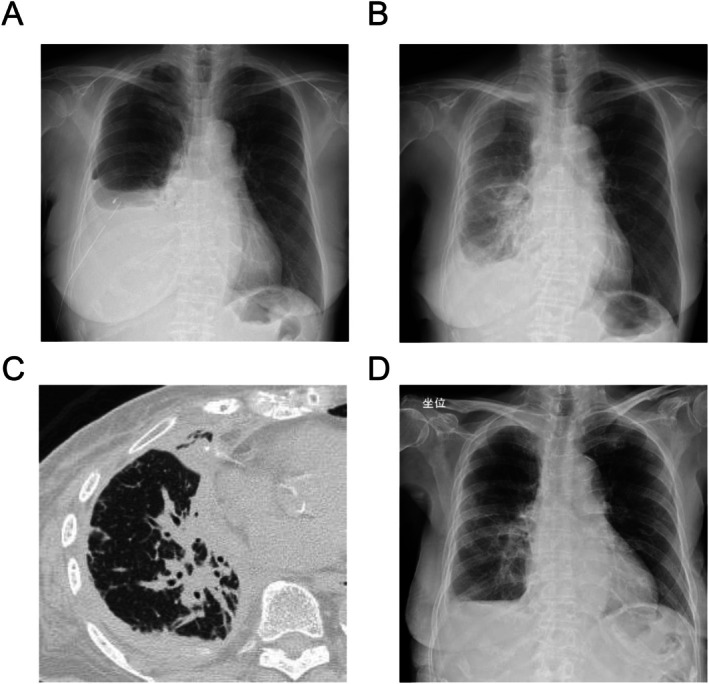
Serial imaging findings (chest radiography and CT). (A) Chest radiography at the time of postoperative recurrence showing a large right pleural effusion consistent with pleural dissemination. (B) Chest radiography obtained immediately before initiation of dabrafenib and trametinib, showing re‐accumulation of right pleural effusion and increased opacity in the right lower lung. (C) Chest CT demonstrating right pleural effusion with perihilar‐predominant infiltrative opacities and bronchial wall thickening in the right lung, findings suggestive of pulmonary lymphangitic carcinomatosis. (D) Chest radiography obtained after initiation of reduced‐dose dabrafenib and trametinib, showing a decrease in right pleural effusion and improvement in lung opacity.

Three years after surgery, imaging studies revealed pleural dissemination, liver metastasis and a large right pleural effusion (Figure 1A). Genetic analysis using the Oncomine Dx Target Test identified a BRAF V600E mutation. Owing to strong concerns regarding treatment‐related adverse events—including pyrexia, fatigue, gastrointestinal symptoms, rash and cardiac dysfunction associated with dabrafenib plus trametinib—and her multiple comorbidities, she declined systemic therapy and opted for best supportive care (BSC).

During BSC, progressive malignant pleural effusion required repeated thoracentesis, followed by chest tube drainage and talc pleurodesis, which temporarily reduced effusion volume. However, chest radiography showed mild re‐accumulation of pleural effusion, and chest CT revealed findings suggestive of pulmonary lymphangitic carcinomatosis (Figure 1B,C), accompanied by worsening dyspnea, pleuritic chest pain and reduced oral intake. As her symptoms markedly impaired her daily functioning and quality of life, the patient re‐evaluated her goals of care. After shared decision‐making with her family and the treating team, she requested initiation of systemic therapy.

Given her advanced age, comorbidities and ECOG PS 2, which were considered to confer a high risk of treatment‐related toxicity, reduced doses of dabrafenib 50 mg twice daily (total 100 mg/day) and trametinib 1 mg once daily were selected based on clinical judgement prioritising safety and treatment continuity, after shared decision‐making with the patient and her family, who preferred treatment initiation at the lowest available dose. Naproxen was administered prophylactically to mitigate the risk of pyrexia. Within 2 weeks, her chest pain resolved, appetite improved and ECOG PS improved from 2 to 1, accompanied by recovery of daily functional capacity and elimination of the need for further pleural drainage procedures and functional improvement sufficient to allow active participation in standing and ambulation rehabilitation. Follow‐up imaging showed reduced pleural effusion (Figure 1D). Serum carcinoembryonic antigen (CEA) markedly decreased from 94.3 ng/mL to approximately 10 ng/mL. No clinically relevant treatment‐related adverse events, including pyrexia, were observed. Dose escalation was considered after initial disease stabilisation; however, it was not pursued due to the favourable clinical response and concern for potential toxicity in this frail patient, and after shared decision‐making with the patient and her family, who expressed strong anxiety about dose escalation given her prior history of drug allergy. Her symptoms remained well controlled, and she maintained stable activities of daily living while continuing therapy under home‐based medical care for more than 6 months.

## 3 Discussion

BRAF V600E–mutant NSCLC is an actionable molecular subset for which combined BRAF/MEK inhibition with dabrafenib plus trametinib has demonstrated clinically meaningful efficacy [1, 2, 3, 4]. However, the regimen is frequently complicated by treatment‐related toxicities, most notably pyrexia, as well as fatigue, gastrointestinal symptoms, rash, edema and laboratory abnormalities, which often result in temporary interruption and subsequent dose modification in real‐world practice [2, 4, 5]. While pivotal trials established the efficacy of this combination, patients with advanced age, multiple comorbidities or poor PS are underrepresented, leaving uncertainty regarding tolerability and optimal dosing strategies in frail populations [2, 4].

This case highlights a practical treatment strategy in frail patients: upfront dose attenuation combined with proactive toxicity management to enable treatment initiation and continuation in individuals who might otherwise be considered unsuitable for systemic therapy. In this context, initiation of dabrafenib and trametinib at reduced doses, together with proactive antipyretic/anti‐inflammatory prophylaxis, led to rapid symptomatic improvement and functional recovery (PS 2 to 1), radiologic reduction of pleural effusion and sustained disease stability for more than 6 months without clinically significant adverse events.

Compared with prior reports in older patients in whom standard‐dose therapy often required early interruption or discontinuation due to pyrexia, this outcome suggests that reduced‐dose initiation may be a feasible approach to improve tolerability while maintaining clinical benefit in carefully selected very elderly or frail patients [5, 6, 7, 8]. From a pharmacological perspective, the preserved efficacy observed with reduced dosing may be partly explained by exposure–response relationships. Dabrafenib is metabolised primarily via CYP3A4 and CYP2C8, and trametinib has a relatively long half‐life, potentially allowing sustained target inhibition even at reduced doses. In elderly patients, age‐related changes in drug clearance and distribution may result in higher systemic exposure, thereby maintaining therapeutic effects despite dose attenuation [9, 10]. Although there are no reports, to our knowledge, describing upfront dose reduction of dabrafenib plus trametinib in patients with poor PS or older patients, similar strategies have been explored with other molecular‐targeted therapies. Low‐dose erlotinib has been reported to be safe and effective in elderly or frail patients with EGFR mutation–positive NSCLC, and targeted therapy has also demonstrated feasibility in patients with poor PS. Therefore, the safety and efficacy of reduced‐dose initiation of dabrafenib plus trametinib may similarly be demonstrated in future studies [11, 12].

Pyrexia is the most frequent toxicity of dabrafenib–trametinib, often necessitating temporary interruption and dose modification [2, 4]. In the present case, naproxen was administered prophylactically to mitigate the risk of pyrexia; however, its contribution cannot be determined from a single case. Management of dabrafenib and trametinib‐related pyrexia syndrome presents significant challenges to treating clinicians, mainly due to the lack of standardised treatment recommendations [13]. While standard pyrexia management typically involves treatment interruption, corticosteroids or antipyretics after symptom onset, prophylactic use of nonsteroidal anti‐inflammatory drugs such as naproxen may represent an anticipatory strategy to prevent early treatment disruption in selected patients. Dose modification is an established management strategy for BRAF/MEK inhibitor–related toxicities, and case‐based evidence indicates that antitumor activity may be preserved after reduction when toxicity limits standard dosing. Although pyrexia management typically involves temporary interruption, stepwise rechallenge and supportive medications, the present case suggests that anticipatory supportive measures combined with conservative starting doses may help avoid early interruption in vulnerable patients, thereby facilitating continued treatment and symptom control [5, 6, 7, 8].

Several limitations should be noted. This report describes a single patient, and the generalizability of reduced‐dose initiation and prophylactic supportive strategies cannot be determined. In addition, the optimal starting dose and supportive regimen remain undefined. Nonetheless, this case adds real‐world evidence that very elderly patients with ECOG PS limitations may still achieve meaningful symptom relief and durable disease control with individualised dosing of dabrafenib and trametinib [5, 6, 7, 8].

In conclusion, an upfront dose‐attenuation strategy combined with proactive toxicity management, including pyrexia prophylaxis, may represent a practical and clinically meaningful approach for selected very elderly or frail patients with BRAF V600E–mutant NSCLC when symptom control and quality of life are major goals. Further accumulation of real‐world data is warranted to clarify patient selection and optimal dose‐modification strategies [2, 4, 5].

## Author Contributions

Shun Suyama, Keeya Sunata and Tetsuo Tani wrote the manuscript. Shun Suyama, Keeya Sunata, Koyuru Uchibori, Shun Shinomiya, Tetsuo Tani and Saeko Takahashi were Involved in clinical management and review of the work.

## Consent

The authors declare that written informed consent was obtained for the publication of this manuscript and accompanying images and attest that the form used to obtain consent from the patient complies with the Journal requirements as outlined in the author guidelines.

## Conflicts of Interest

The authors declare no conflicts of interest.

## Data Availability

Data sharing not applicable to this article as no datasets were generated or analysed during the current study.
